# Orally administered *Lactobacillus rhamnosus* modulates the respiratory immune response triggered by the viral pathogen-associated molecular pattern poly(I:C)

**DOI:** 10.1186/1471-2172-13-53

**Published:** 2012-09-18

**Authors:** Julio Villena, Eriko Chiba, Yohsuke Tomosada, Susana Salva, Gabriela Marranzino, Haruki Kitazawa, Susana Alvarez

**Affiliations:** 1 Laboratory of Clinical and Experimental Biochemistry, Reference Centre for Lactobacilli (CERELA-CONICET), Tucuman, Argentina; 2 Food Immunology Group, Graduate School of Agricultural Science, Tohoku University, Sendai, 981-8555, Japan

**Keywords:** *L. rhamnosus* CRL1505, Poly(I:C), Antiviral immunity, Respiratory tract

## Abstract

**Background:**

Some studies have shown that probiotics, including *Lactobacillus rhamnosus* CRL1505, had the potential to beneficially modulate the outcome of certain bacterial and viral respiratory infections. However, these studies did not determine the mechanism(s) by which probiotics contribute to host defense against respiratory viruses.

**Results:**

In this work we demonstrated that orally administered *Lactobacillus rhamnosus* CRL1505 (Lr1505) was able to increase the levels of IFN-γ, IL-10 and IL-6 in the respiratory tract and the number of lung CD3^+^CD4^+^IFN-γ^+^ T cells. To mimic the pro-inflammatory and physiopathological consecuences of RNA viral infections in the lung, we used an experimental model of lung inflammation based on the administration of the artificial viral pathogen-associated molecular pattern poly(I:C). Nasal administration of poly(I:C) to mice induced a marked impairment of lung function that was accompanied by the production of pro-inflammatory mediators and inflammatory cell recruitment into the airways. The preventive administration of Lr1505 reduced lung injuries and the production of TNF-α, IL-6, IL-8 and MCP-1 in the respiratory tract after the challenge with poly(I:C). Moreover, Lr1505 induced a significant increase in lung and serum IL-10. We also observed that Lr1505 was able to increase respiratory IFN-γ levels and the number of lung CD3^+^CD4^+^IFN-γ^+^ T cells after poly(I:C) challenge. Moreover, higher numbers of both CD103^+^ and CD11b^high^ dendritic cells and increased expression of MHC-II, IL-12 and IFN-γ in these cell populations were found in lungs of Lr1505-treated mice. Therefore, Lr1505 treatment would beneficially regulate the balance between pro-inflammatory mediators and IL-10, allowing an effective inflammatory response against infection and avoiding tissue damage.

**Conclusions:**

Results showed that Lr1505 would induce a mobilization of cells from intestine and changes in cytokine profile that would be able to beneficially modulate the respiratory mucosal immunity. Although deeper studies are needed using challenges with respiratory viruses, the results in this study suggest that Lr1505, a potent inducer of antiviral cytokines, may be useful as a prophylactic agent to control respiratory virus infection.

## Background

Studies in animal models have identified strong immunomodulatory effects of some non-pathogenic bacteria and provided evidence that some orally administered lactic acid bacteria (LAB) strains can activate the common mucosal immune system and, thus, influence sites distant to the intestine, including the respiratory tract [1,2]. Respiratory effects of probiotics in animal models have included attenuating allergic airway responses and protecting against respiratory pathogens [1,2]. Moreover, several human trials have demonstrated that probiotics, when taken prophylactically by healthy individuals induce a beneficial effect on the severity and duration of bacterial and viral respiratory infections reviewed in [3].

In this sense, our laboratory evaluated several *Lactobacillus* strains isolated from goat’s milk according to their capacity to modulate the mucosal immune system and found that the oral administration of *Lactobacillus rhamnosus* CRL1505 (Lr1505) was able to improve respiratory immunity [4]. We observed that Lr1505 induced an increase in the levels of interleukin (IL)-6, IL-4, IL-10 and interferon (IFN)-γ in broncho-alveolar lavage fluid (BAL) of treated mice. Moreover, Lr1505, administered by the oral route at the proper dose, was able to increase *Streptococcus pneumoniae* clearance rates in lung and blood, improved survival of infected mice and reduced lung injuries [4,5]. We also conducted experiments aimed to evaluate the effect of Lr1505 on the health of children attending pre-school dining community centers. We evaluate the impact of probiotic yogurt containing Lr1505 on mucosal immunity and we study the effect of this treatment on gastrointestinal and respiratory infections, in terms of number of episodes and severity [6,7]. Significant differences were detected in the incidence of intestinal and respiratory infections, especially those caused by viruses, when the placebo and the probiotic treatments were compared. These results indicate that Lr1505 can improve resistance not only against bacterial respiratory pathogens but to respiratory viruses as well [7].

Among respiratory viruses, influenza A virus and respiratory syncytial virus (RSV) are the most important cause of infant lower respiratory tract infection, causing significant morbidity and mortality especially in developing countries [8,9]. Although genetically dissimilar, both viruses generate double-stranded (ds) RNA replication intermediates that act as toll-like receptor 3 (TLR3) and retinoic acid-inducible gene I (RIG-I) ligands and contribute to immune system activation. Influenza A virus, a single-stranded RNA virus has been shown to trigger type I IFN through recognition by TLR3 and RIG-I in myeloid dendritic cells (DCs), fibroblasts or alveolar epithelial cells [10]. In addition, TLR3 expressed by respiratory epithelial cells and DCs contributes at recognizing RSV during infection by binding to viral RNA [11].

Previous *in vitro* studies have shown that treatment of murine or human primary respiratory epithelial cells or cell lines with poly(I:C) induces secretion of multiple chemokines, particularly monocyte chemotactic protein (MIP)-1, RANTES and IL-8, together with increased expression of genes encoding TLRs, including TLR3 [12]. *In vivo* studies using mice have demonstrated that poly(I:C) treatment results in TLR3- and CXCR2-dependent neutrophilic pulmonary inflammation, interstitial edema, bronchiolar epithelial hypertrophy, and altered lung function [13,14]. These changes were accompanied by elevated levels of pro-inflammatory cytokines and type I interferons in BAL [13] and, increased airway epithelial cell TLR3 protein expression [14]. These functional changes are similar to those caused by RSV and therefore, nasal administration of the dsRNA analog poly(I:C) has been used to mimic the pro-inflammatory and physiopathological consecuences of RNA viral infections in the lung.

As mentioned above, some studies have shown that probiotics, including Lr1505, had the potential to beneficially modulate the outcome of certain bacterial and viral respiratory infections [3]. However, these studies did not determine the mechanism(s) by which immunobiotics contribute to host defense against respiratory viruses. An understanding of how the dialogue between immunobiotics and the innate immune system is translated into beneficial/protective responses is required before we can achieve clinically effective bacteria-based strategies that maintain and promote respiratory health. In this sense, studying the effect of orally administered probiotics on the immune response triggered by respiratory activation of TLR3/RIG-I would contribute to the knowledge of the mechanism of probiotics’ protective effect against respiratory viral infections. Therefore, the aim of the present study was to deepen the understanding of the mechanisms of Lr1505 immunomodulatory activity by evaluating: a) its effects on immune cell populations in gut and lung; b) its ability to change the cytokine profile in serum and the respiratory tract; c) and its influence on the respiratory immune response induced by intranasal challenge with the viral pathogen-associated molecular pattern poly(I:C).

## Methods

### Microorganisms

*Lactobacillus rhamnosus* CRL1505 (Lr1505) and CRL1506 (Lr1506) were obtained from the CERELA culture collection (Chacabuco 145, San Miguel de Tucumán, Argentina). Both strains were isolated from goat milk from northwestern Argentina and were selected because their immunomodulatory capacities [4]. The culture was kept freeze-dried and then rehydrated using the following medium: peptone 15.0 g, tryptone 10.0 g, meat extract 5.0 g, distilled water 1 l, pH 7. It was cultured for 12 h at 37°C (final log phase) in Man-Rogosa-Sharpe broth (MRS, Oxoid). The bacteria were harvested by centrifugation at 3000 g for 10 min, washed three times with sterile 0.01 mol/l phosphate buffer saline (PBS), pH 7.2, and resuspended in sterile 10% non-fat milk.

### Animals and feeding procedures

Male 6-week-old BALB/c mice were obtained from the closed colony kept at CERELA. They were housed in plastic cages at room temperature. Mice were housed individually during the experiments and the assays for each parameter studied were performed in 5–6 mice per group for each time point. Lr1505 or Lr1506 was administered to different groups of mice for 5 consecutive days at a dose of 10^8^ cells/mouse/day in the drinking water [4,5]. The treated groups and the untreated control group were fed a conventional balanced diet *ad libitum*. All experiments were carried out in compliance with the Guide for Care and Use of Laboratory Animals and approved by the Ethical Committee of Animal Care at CERELA under the protocol BIOT-CRL/11.

### Intransal administration of poly(I:C)

Mice were lightly anesthetized and 100 μl of PBS, containing 250 μg poly(I:C) (equivalent to 10 mg/kg body weight), was administered dropwise, via the nares. Control animals received 100 μl of PBS. Mice received three doses of poly(I:C) or PBS with 24 hs rest period between each administration.

### Cytokine concentrations in serum, broncho-alveolar and intestinal fluids

Blood samples were obtained through cardiac puncture at the end of each treatment and collected in heparinized tubes. BAL samples were obtained as described previously [15]. Briefly, the trachea was exposed and intubated with a catheter, and 2 sequential bronchoalveolar lavages were performed in each mouse by injecting sterile PBS; the recovered fluid was centrifuged for 10 min at 900 x g; the pellet was used to make smears that were stained for cell counts; and the fluid was frozen at −70°C for subsequent antibody analyses. Intestinal fluid samples were obtained as follows: the small intestine was flushed with 5 ml of PBS and the fluid was centrifuged (10,000 g, 4°C 10 min) to separate particulate material. The supernatant was kept frozen until use. Tumour necrosis factor (TNF)-α, IFN-α, IFN-β, IFN-γ, IL-4, IL-6, IL-8, IL-10, IL-12 and MCP-1 concentrations in serum, BAL and intestinal fluid, were measured with commercially available enzyme-linked immunosorbent assay (ELISA) technique kits following the manufacturer's recommendations (R&D Systems, MN, USA) [4].

### Leukocyte counts in the blood and BAL

Blood and BAL samples were obtained as described above. The total number of leukocytes and differential cell counts were performed as described previously [15]. Briefly, the total number of leukocytes was determined with a hemocytometer. Differential cell counts were performed by counting 200 cells in blood smears stained with May Grunwald-Giemsa.

### Biochemical assay of BAL fluid

Protein and albumin content, a measure to quantitate increased permeability of the bronchoalveolar–capillarity barrier, and lactate dehydrogenase (LDH) activity, an indicator of general cytotoxicity, were determined in the acellular BAL fluid [15]. Protein content was measured by the bicinchoninic (BCA) protein assay (Pierce Biotechnology Inc., Rockford, IL). Albumin content was determined colorimetrically based on albumin binding to bromcresol green using an albumin diagnostic kit (Wiener Lab, Buenos Aires, Argentina). LDH activity, expressed as units per liter of BAL fluid, was determined by measuring the formation of the reduced form of nicotinamide adenine dinucleotide (NAD) using the Wiener reagents and procedures (Wiener Lab).

### Lung wet:dry weight ratio

Lung wet:dry weight ratio was measured as previously described by Aeffner et al., [16]. Briefly, mice were euthanized and exsanguinated, and their lungs removed, weighed, and dried in an oven at 55°C for 7 days. After drying, the lungs were weighed again. Wet:dry weight ratio was then calculated as an index of intrapulmonary fluid accumulation, without correction for blood content.

### Cell preparation

Single Peyer’s patches (PPs) and lung cells from mice were prepared using the method described by Hori et al. [17]. Mice were anaesthetized with diethyl ether and killed the next day by exsanguination. Lungs were removed, finely minced and incubated for 90 min with 300 U of collagenase (Yakult Honsha Co., Tokyo, Japan) in 15 ml of RPMI 1640 medium (Sigma, Tokyo, Japan). To dissociate the tissue into single cells, collagenase-treated minced lungs were gently tapped into a plastic dish. After removal of debris, erythrocytes were depleted by hypotonic lysis. The cells were washed with RPMI medium supplemented with 100 U/ml of penicillin and 100 mg/ml of streptomycin and then resuspended in a medium supplemented with 10% heat-inactivated foetal calf serum (FCS). Cells were counted using Trypan Blue exclusion and then resuspended at an appropriate concentration of 5x10^6^ cells/ml.

### Flow cytometry studies

Lung cell suspensions were pre-incubated with anti-mouse CD32/CD16 monoclonal antibody (Fc block) for 15 min at 4°C. Cells were incubated in the antibody mixes for 30 min at 4°C and washed with FACS buffer. The following antibodies from BD PharMingen were used: anti-mouse CD3-FITC, anti-mouse CD4-PE, anti-mouse CD8-PE, anti-mouse IFN-γ-APC, anti-mouse CD11b-FITC, anti-mouse CD11c-PE, anti-mouse IFN-γ-PE, anti-mouse MHC-II-PE, anti-mouse IL-12-PE and anti-mouse CD103-biotin. Following incubation with biotinylated primary antibodies, the labeling was revealed using streptavidin-PercP. In all cases, cells were then acquired on a BD FACSCalibur^™^ flow cytometer (BD Biosciences) and data were analyzed with FlowJo software (TreeStar). The total number of cells in each population was determined by multiplying the percentages of subsets within a series of marker negative or positive gates by the total cell number determined for each tissue.

### Statistical analysis

Experiments were performed in triplicate and results were expressed as mean ± standard deviation (SD). After verification of the normal distribution of data, 2-way ANOVA was used. Tukey's test (for pairwise comparisons of the means) was used to test for differences between the groups. Differences were considered significant at p < 0.05.

## Results

### L. rhamnosus CRL1505 and L. rhamnosus CRL1506 differentially modulate intestinal immunity

**Figure 1:**
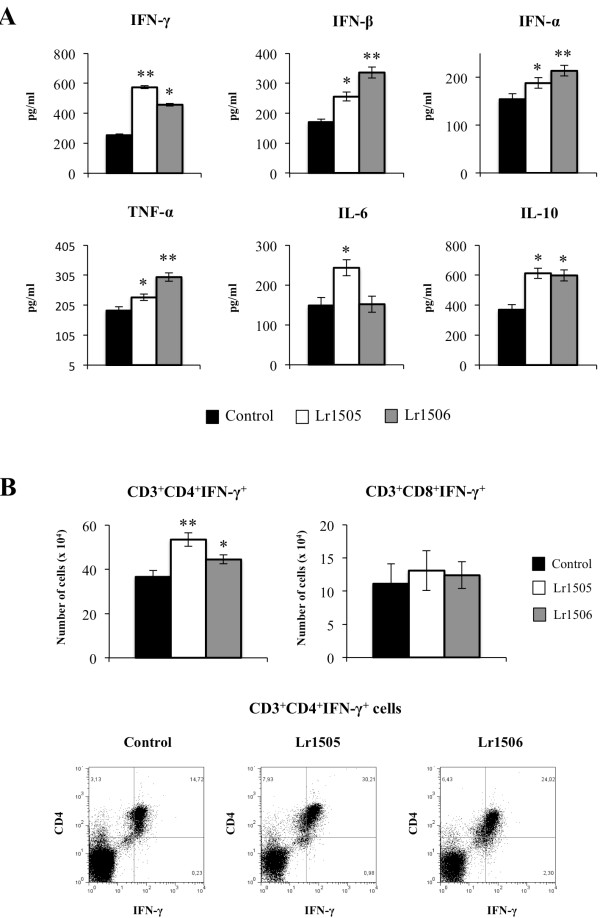
**Effect of lactobacilli on intestinal immunity.** (**A**) Effect of *Lactobacillus rhamnosus* CRL1505 (Lr1505) or *L. rhamnosus* CRL1506 (Lr1506) administration on the tumor necrosis factor (TNF)-α, interferon (IFN)-α, IFN-β, IFN-γ, interleukin (IL)-6, and IL-10 concentrations in intestinal fluid. (**B**) Effect of Lr1505 or Lr1506 administration on CD3^+^CD8^+^IFN-γ^+^ and CD3^+^CD4^+^IFN-γ^+^ T cells from Peyer’s patches. The results represent data from three independent experiments. Significant differences between lactobacilli-treated and control groups * (*P* < 0.05) or ** (*P* < 0.01).

The immunomodulatory effects of Lr1505 and Lr1506 were determined *in vivo* using BALB/c mice. The administration of both strains induced significant changes in the profile of cytokines in the intestinal fluid. The levels of IFN-α, IFN-β, IFN-γ and TNF-α were higher in animals treated with Lr1505 or Lr1506 than in controls (Figure 1A). Levels of TNF-α and type I interferons were higher in Lr1506-treated mice than in the other experimental groups, while Lr1505 strain was more effective than Lr1506 for improving production of IFN-γ (Figure 1A). On the other hand, only Lr1505 was able to increase the levels of intestinal IL-6 (Figure 1A). When studying the regulatory cytokines IL-10 and TGF-β we observed that both strains were able to increase the levels of IL-10 (Figure 1A), while lactobacilli treatments did not modified the values of TGF-β (data not shown).

**Figure 2:**
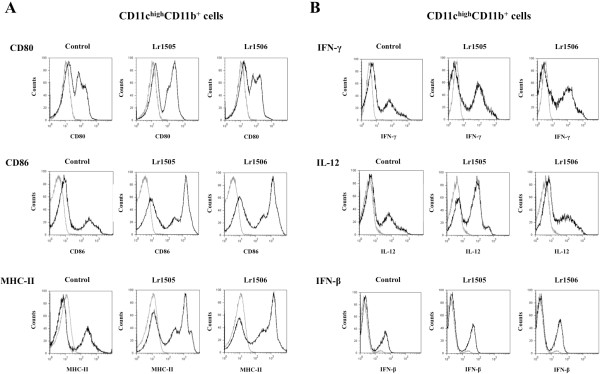
**Effect of lactobacilli on intestinal immunity.** Effect of *Lactobacillus rhamnosus* CRL1505 (Lr1505) or *L. rhamnosus* CRL1506 (Lr1506) administration on CD11c^high^CD11b^+^ dendritic cells from Peyer’s patches. Expression of MHC-II, CD80, CD86 (**A**) and IFN-γ, IL-12 and IFN-β (**B**) in CD11c^high^CD11b^+^ cells. The results represent data from three independent experiments.

We also evaluated the changes induced by lactobacilli in immune cell populations from PPs. Both Lr1505 and Lr1506 increased the numbers of CD3^+^CD4^+^IFN-γ^+^ T cells (Figure 1B). On the contrary, neither Lr1505 nor Lr1506 induced changes in the number of CD3^+^CD8^+^IFN-γ^+^ T cells (Figure 1B). When we studied the effect of lactobacilli on CD11c^high^CD11b^+^ dendritic cells (DCs) from PPs, we observed no changes in the number of this population (data not shown). However, both Lr1505 and Lr1506 strains increased the expression of MHC-II and CD80 and CD86 co-stimulatory molecules (Figure 2A). We also observed increased levels of IFN-γ and IL-12 in CD11c^high^CD11b^+^ DCs from Lr1505 treated mice, while none of the strains modify the levels of IFN-β (Figure 2B).

### L. rhamnosus CRL1505 but not L. rhamnosus CRL1506 modulates respiratory immunity

**Figure 3:**
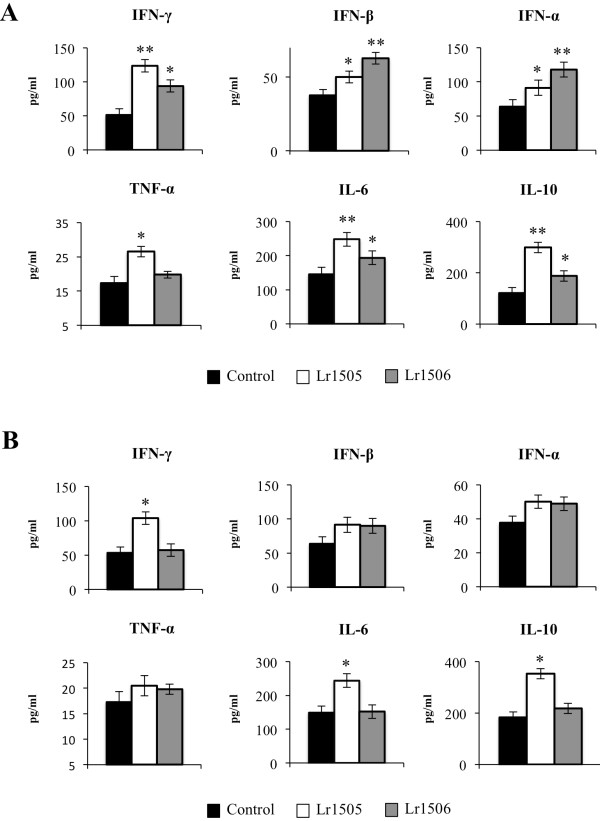
**Effect of lactobacilli on systemic and respiratory immunity.** Effect of *Lactobacillus rhamnosus* CRL1505 (Lr1505) or *L. rhamnosus* CRL1506 (Lr1506) administration on the tumor necrosis factor (TNF)-α, interferon (IFN)-α, IFN-β, IFN-γ, interleukin (IL)-6, and IL-10 concentrations in serum (**A**) and broncho-alveolar lavages (**B**). The results represent data from three independent experiments. Significant differences between lactobacilli-treated and control groups * (*P* < 0.05) or ** (*P* < 0.01).

We have previously shown that changes in the profile of cytokines induced in intestine by LAB strains are reflected in the blood [4]. Therefore, we also assessed the levels of different cytokines in serum samples from animals treated with Lr1505 or Lr1506 and controls. As shown in Figure 3A, both strains increased the levels of type I interferons, IFN-γ, IL-6 and IL-10 whereas no significant differences were observed in the levels of IL-8 and MCP-1 (data not shown). Lr1506 administration was more effective than Lr1505 to increase serum levels of IFN-α and-β, whereas strain Lr1505 induced higher levels of IFN-γ, IL-6 and IL-10 (Figure 3A). In addition, only Lr1506 was able to increase serum TNF-α (Figure 3A).

**Figure 4:**
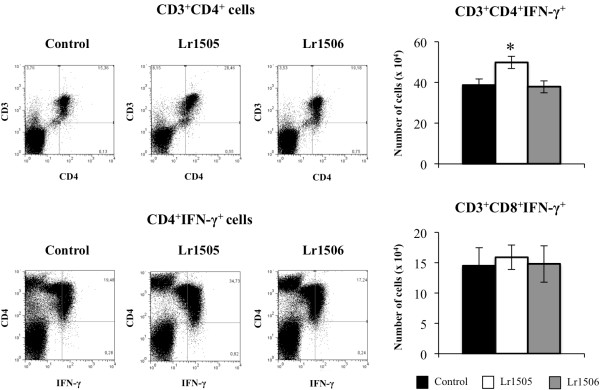
**Effect of lactobacilli on respiratory immunity.** Effect of *Lactobacillus rhamnosus* CRL1505 (Lr1505) or *L. rhamnosus* CRL1506 (Lr1506) administration on CD3^+^CD8^+^IFN-γ^+^ and CD3^+^CD4^+^IFN-γ^+^ T cells from lung. The results represent data from three independent experiments. Significant differences between lactobacilli-treated and control groups * (*P* < 0.05).

In order to evaluate the changes induced by LAB in respiratory tract we determined the levels of different cytokines in BAL (Figure 3B). Lr1506 treatment induced no changes in any of the cytokines evaluated. On the contrary, oral administration of Lr1505 induced increases in levels of IL-6, IFN-γ and IL-10 in BAL (Figure 3B). We also evaluated the changes induced by both strains in lung immune cells using flow cytometry. Orally administered Lr1505 was able to increase the number of CD3^+^CD4^+^IFN-γ^+^ T cells in lungs while no modifications were observed in the number of CD3^+^CD8^+^IFN-γ^+^ T cells (Figure 4). Furthermore, Lr1506 was not able to induce changes in the number of lung T cells. In lungs, two populations of DCs can be defined using CD11c, CD11b, CD103 and MHC-II antibodies: MHC-II^+^CD11c^+^CD11b^low^CD103^+^ and MHC-II^+^CD11c^+^CD11b^high^CD103^-^ cells [17]. Therefore, we next aimed to evaluate the effect of LAB on these populations of DCs from lungs. Lactobacilli did not induce changes in the number of lung CD11c^+^CD11b^low^CD103^+^ and CD11c^+^CD11b^high^CD103^-^ DCs or modify the expression of MHC-II in these DCs (data not shown).

### Poly(I:C) induces pulmonary injuries and dysfunction that are reduced by L. rhamnosus CRL1505 administration

Considering the ability of Lr1505 to stimulate respiratory tract immunity and our previous studies in humans demonstrating that the administration of this probiotic strain is able increase resistance to respiratory viral infections in children [7], we next evaluated the effect of Lr1505 on the immune response triggered by nasal administration of the viral pathogen-associated molecular pattern poly(I:C).

**Figure 5:**
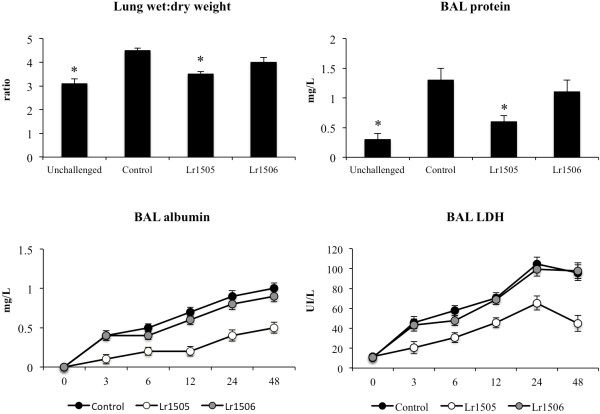
**Effect of lactobacilli on lung injuries induced by the nasal administration of the viral pathogen-associated molecular pattern poly(I:C).** Effect of *Lactobacillus rhamnosus* CRL1505 (Lr1505) or *L. rhamnosus* CRL1506 (Lr1506) administration on lung wet:dry weight ratio, lactate dehydrogenase (LDH) activity and, albumin and protein concentrations in broncho-alveolar lavages (BAL). The results represent data from three independent experiments. Significant differences between lactobacilli-treated and control groups * (*P* < 0.05).

Previous studies from Aeffner et al., [16] demonstrated that the nasal challenge of mice with poly(I:C) significantly alters lungs function and induce lung injuries. Our results are in line with that study since we observed altered wet:dry weight ratio in poly(I:C)-challlenged mice (Figure 5). Moreover, significantly increased levels of protein and albumin concentrations as well as LDH activity was found in BAL samples of challenged mice indicating that poly(I:C) produces an alteration of the alveolar-capillary barrier and local cellular damage. Lr1505 treatment decreased significantly the parameters that we use to evaluate pulmonary damage, whereas Lr1506-treated mice showed lung injuries similar to those observed in the control group (Figure 5).

### L. rhamnosus CRL1505 administration beneficially modulates immune response triggered by nasal administration of poly(I:C)

**Figure 6:**
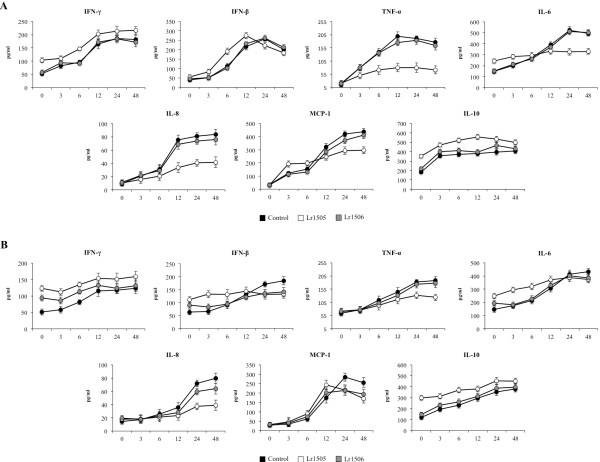
**Effect of lactobacilli on the production of cytokines induced by the nasal administration of the viral pathogen-associated molecular pattern poly(I:C).** Effect of *Lactobacillus rhamnosus* CRL1505 (Lr1505) or *L. rhamnosus* CRL1506 (Lr1506) administration on the tumor necrosis factor (TNF)-α, interferon (IFN)-β, IFN-γ, interleukin (IL)-6, IL-8, IL-10 and monocyte chemotactic protein (MCP)-1 concentrations in serum (**A**) and broncho-alveolar lavages (**B**). The results represent data from three independent experiments.

Lung tissue injuries induced by the nasal administration of poly(I:C) has been associated to an exacerbated inflammatory response [14,16]. Therefore, we next evaluated the pulmonary immune response induced by the nasal challenge with poly(I:C) and the effect of Lr1505 in that response. First, we studied the levels of IFN-α, IFN-β, IFN-γ, IL-6, IL-4, TNF-α, IL-1β, IL-8, MCP-1, IL-10 and TGF-β in BAL and serum samples at different hours after poly(I:C) challenge (Figure 6). Nasal administration poly(I:C) significantly increased respiratory levels of pro-inflammatory mediators IL-6, TNF-α, IL-1β, IL-8 and MCP-1. No differences were observed between the experimental groups when analyzing IL-1β levels (data not shown). However, levels of IL-6, TNF-α, IL-8 and MCP-1 were significantly higher in the Lr1505 group when compared to controls (Figure 6A). IFN-α, IFN-β and IFN-γ in BAL were also increased after the challenge with poly(I:C) in all the experimental groups, however, Lr1505 mice showed higher levels of BAL IFN-β and IFN-γ than controls (Figure 6A). No changes were observed in BAL IL-4 concentration during the studied period (data not shown). Poly(I:C) challenge also induced an increase in the respiratory levels of IL-10 and TGF-β in all groups. Lr1505 treated mice presented levels of TGF-β that were similar to those in controls (data not shown). However, levels of IL-10 were significantly higher in Lr1505 treated mice (Figure 6A). Oral administration of Lr1506 did not induce changes in the levels of respiratory cytokines (Figure 6A).

The nasal challenge with poly(I:C) also increased cytokines levels in serum. Moreover, the effect of Lr1505 treatment on the production of these cytokines was similar to that found in BAL (Figure 6B). Lr1505 induced increases in serum IFN-β, IFN-γ and IL-10, while decreased production of TNF-α, IL-8 and MCP-1 when compared to controls (Figure 6B). In addition, Lr1505 induced higher levels of serum IL-6 (Figure 6B).

**Figure 7:**
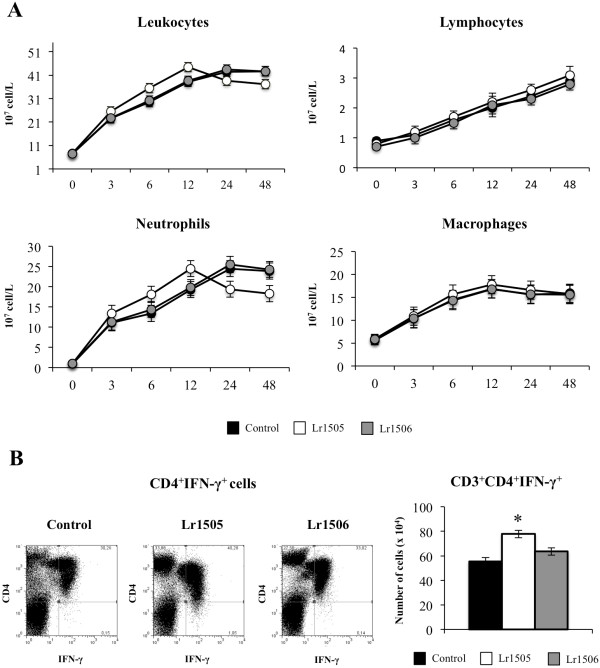
**Effect of lactobacilli on lung immune cells recruitment induced by the nasal administration of the viral pathogen-associated molecular pattern poly(I:C).** Effect of *Lactobacillus rhamnosus* CRL1505 (Lr1505) or *L. rhamnosus* CRL1506 (Lr1506) administration on the number of leukocytes, lymphocytes, neutrophils and macrophages after the challenge with poly(I:C) (**A**). Effect of Lr1505 or Lr1506 administration on CD3^+^CD4^+^IFN-γ^+^ T cells from lung after the challenge with poly(I:C) (**B**). The results represent data from three independent experiments. Significant differences between lactobacilli-treated and control groups * (*P* < 0.05).

**Figure 8:**
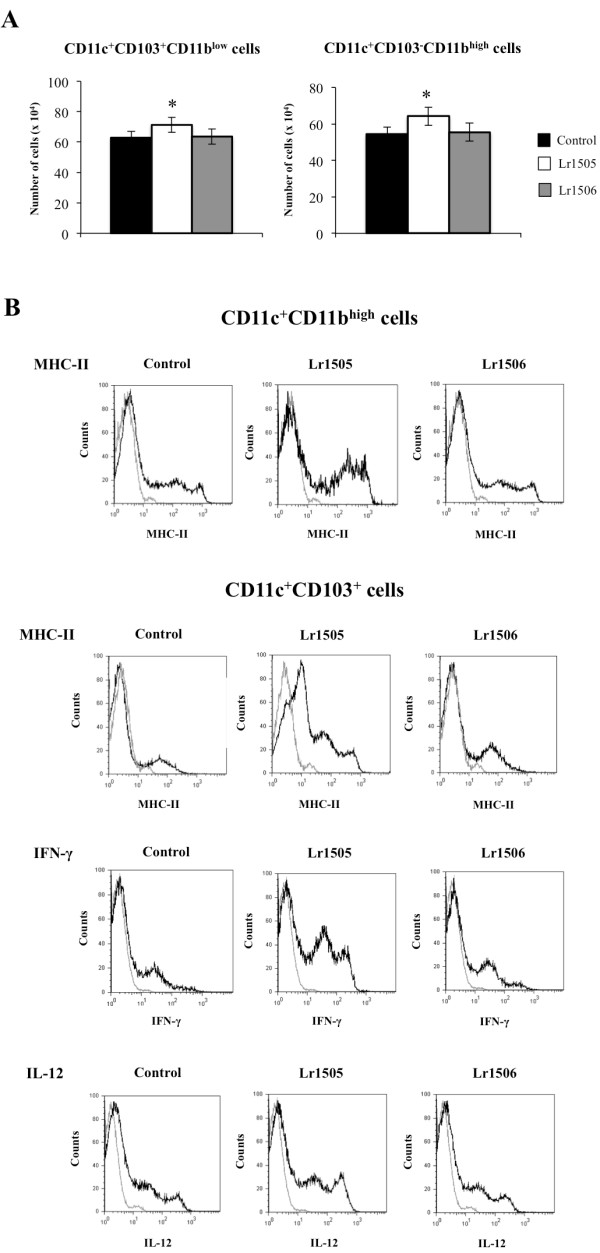
**Effect of lactobacilli on lung dendritic cells activation induced by the nasal administration of the viral pathogen-associated molecular pattern poly(I:C).** Effect of *Lactobacillus rhamnosus* CRL1505 (Lr1505) or *L. rhamnosus* CRL1506 (Lr1506) administration on the number of pulmonary CD11c^+^CD11b^low^CD103^+^ and CD11c^+^CD11b^high^CD103^-^ dendritic cells after the challenge with poly(I:C) (**A**). Effect of Lr1505 or Lr1506 administration on the expression of MHC-II, IFN-γ and IL-12 in pulmonary CD11c^+^CD11b^low^CD103^+^ and CD11c^+^CD11b^high^CD103^-^ dendritic cells after the challenge with poly(I:C) (**B**). The results represent data from three independent experiments. Significant differences between lactobacilli-treated and control groups * (*P* < 0.05).

We also evaluated the changes in lung immune cells induced by nasally administered poly(I:C) (Figures 7, 8). The numbers of total infiltrated cells, macrophages, neutrophils and lymphocytes increased in a time-dependent manner in all the experimental groups. The number of neutrophils in Lr1505 treated mice present significant differences with respect to the controls, showing higher values during the first hours and then decreased numbers of cells by the end of the studied period (Figure 7A). In addition, the number of BAL lymphocytes was superior in Lr1505 treated mice when compared to controls after hour 12 post-challenge (Figure 7A). Poly(I:C) administration increased CD3^+^CD8^+^IFN-γ^+^ (data not shown) and CD3^+^CD4^+^IFN-γ^+^ T cells (Figure 7B). However, Lr1505 treatment induced significantly higher levels of lung CD3^+^CD4^+^IFN-γ^+^ T cells when compared to controls (Figure 7B). Poly(I:C) challenge also increased the number of pulmonary CD11c^+^CD11b^low^CD103^+^ and CD11c^+^CD11b^high^CD103^-^ DCs when compared to basal levels in all the experimental groups (Figure 8A). Oral administration of Lr1505 significantly increased the numbers of both populations of DCs cells in lungs when compared to controls (Figure 8A). Moreover, this treatment improved expression of MHC-II in both CD11c^+^CD11b^low^CD103^+^ and CD11c^+^CD11b^high^CD103^-^ lung DCs. However, production of IL12 and IFN-γ was improved only in CD11c^+^CD11b^low^CD103^+^ cells (Figure 8B). On the contrary, no modifications in pulmonary T cells and DCs were observed after oral treatment with Lr1506 when compared to controls (Figures 7, 8).

## Discussion

Several works have described different effects of probiotics on intestinal immune system from attenuating inflammatory responses to improving immunity [18-20]. Previously, our laboratory evaluated the effect of the oral administration of two Lactobacillus strains of the same origin and with similar technological properties on the production of IFN-γ, IL-4 and IL-10 in the intestine and we demonstrated that Lr1505 and Lr1506 were able to induce differential cytokine profiles in the gut [4]. The first aim of our present research was to further evaluate the changes induced by Lr1505 and Lr1506 in intestinal immunity. *In vivo* experiments demonstrated that the administration of lactobacilli strains significantly augmented the expression of IFN-γ in PPs compared with the control, confirming our previous results [4]. Moreover, Lr1505 was more efficient than Lr1506 for increasing the levels of IFN-γ, IL-10 and IL-6 in the intestine. It is well established that a high IL-12 production of DCs by microbial stimuli gives rise to Th1 polarization and thus a strong stimulation of the adaptive immune defense. In fact, oral administration of LAB to mice has been reported to augment IL-12 and IFN-γ mRNA expressions and CD4^+^ T cell-DCs interaction in PPs [21]. Studies showed that probiotics are captured by CD11c^+^ DCs in PPs and increase IL-12 production by these antigen presenting cells. Subsequently, T cells receive the information from DCs, resulting in the immune activation of CD4^+^ T and increased production of IL-6 and IFN-γ [22]. Therefore, Lr1505 would be able to improve intestinal Th1 immune response through this mechanism and it would be more efficient than Lr1506.

**Figure 9:**
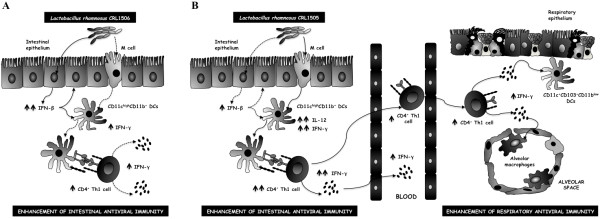
**Proposed mechanism for the improvement of antiviral immunity by orally administered lactobacilli.** Enhancement of intestinal antiviral immunity by *Lactobacillus rhamnosus* CRL1506 (**A**). Enhancement of intestinal and respiratory antiviral immunity by *Lactobacillus rhamnosus* CRL1505 (**B**). Orally administered *L. rhamnosus* CRL1505 stimulates the Th1 response in the gut and to induce mobilization of Th1 cells from inductive sites in the gut to effector sites in the respiratory tract. These activated Th1 cells would produce cytokines (IFN-γ) able to stimulate the activity of local respiratory immune cells such as alveolar macrophages, NK cells and CD11c^+^CD11b^low^CD103^+^ dendritic cells. These previously activated immune cells would be able to efficiently phagocyte pathogens that reach the alveolar space, induce specific immune responses and increase the resistance to bacterial and viral respiratory infections.

On the contrary, Lr1506 showed a higher capacity to improve levels of IFN-α, IFN-β and TNF-α in the gut when compared with Lr1505. It was observed that certain lactobacilli trigger the expression of IFN-β in DCs cells [23]. In our present analyses, we therefore expected to find that Lr1506 was capable of increasing IFN-β levels in DCs; however, we did not detected changes of this cytokine in CD11c^high^CD11b^+^ cells from treated mice. Therefore, the increased levels of intestinal IFN-β observed in our *in vivo* experiments could indicate that the production of this cytokine is in charge of intestinal epithelial cells (IEC). In this sense, we have evaluated the effect of different LAB strains on bovine and porcine IEC and we found that different LAB strains had distinct effects on cytokine production by these cells. Notably, some strains such as *L. casei* MEP221106 and *L. rhamnosus* LA-2 were able to increase IFN-β production in IEC [20,24]. Moreover, we also evaluated the response of IEC to poly(I:C) challenge and found that *L. casei* MEP221106 and *L. rhamnosus* LA-2 improved the levels of IFN-α, IFN-β and TNF-α in porcine and bovine IEC respectively [20,24]. Considering that IFN-β gives rise to the up-regulation of a vast number of genes involved in viral defense but also genes of major importance for the development of a strong cellular (Th1) response, including the expression of IL-12 and CXCL10, we can speculate that Lr1506 may play an important role in the improvement of innate and specific immune responses against intestinal virus. In addition, our results demonstrated that Lr1505 and Lr1506 have the ability to improve intestinal antiviral immunity by using different mechanisms (Figure 9).

When we evaluated the levels of serum cytokines we found that Lr1506 was more efficient than Lr1505 to increase IFN-α, IFN-β and TNF-α, while serum IFN-γ, IL-10 and IL-6 levels were more efficiently improved by Lr1505. These changes in the profile of serum cytokines was similar to those found in the intestinal fluid, indicating that levels of serum cytokines are a reflection of intestinal changes and confirming our previous findings in this regard (4). On the contrary, the analysis of respiratory cytokines showed that only Lr1505 was able to increase the levels of IFN-γ, IL-10 and IL-6. While these are the same cytokines that were increased by this strain in serum, we can not attribute a direct correlation between the two increases, as we did not found increased levels of IFN-α, IFN-β or TNF-α in the respiratory tract of Lr1506 treated mice. Therefore, and taking into account the capacity of Lr1505 of increasing the number of CD3^+^CD4^+^IFN-γ^+^ T cells in PPs, we hypothesized that Lr1505 would be able to induce a mobilization of these cells into the respiratory mucosa. We demonstrated that this hypothesis was true since increased numbers of CD3^+^CD4^+^IFN-γ^+^ T were found in lungs of Lr1505 treated mice. Considering that several studies reported that oral administration of probiotic strains increased protection against influenza virus infection in mice by increasing NK cell activity and IFN-γ production in lung [17,21,25], we can speculate that the mobilization of CD3^+^CD4^+^IFN-γ^+^ T cells from the intestine to the airways and the improved production of IFN-γ could be involved in the protective effect against viral infections induced by Lr1505 that was observed in clinical studies [7]. Moreover, the increased levels of serum IFN-β induced by this strain could also involved in its protective effect since it was demonstrated that the oral administration of *L. plantarum* L-137 enhanced protection against influenza virus infection in correlation with an increase in IFN-β production in the serum of infected mice at an early stage after infection [26].

To mimic the pro-inflammatory and physiopathological consecuences of RNA viral infections in the lung, we used an experimental model of lung inflammation based on the administration of the artificial TLR3/RIG-I ligand and dsRNA analog poly(I:C). In our experiments, administration of poly(I:C) to the lungs of mice induced a marked impairment of lung function that was accompanied by the production of pro-inflammatory mediators and inflammatory cell recruitment into the airways in accordance with results published by Stowell et al. [14]. Exposure to poly(I:C) induced respiratory epithelial cell death and impaired epithelial barrier function as demonstrated by the increased levels LDH activity and albumin concentration in BAL. Moreover, intranasal administration of three once-daily doses of poly(I:C) resulted in an inflammatory cell influx into the lung. This increase in total cellularity in the BAL samples was due to a significant influx of neutrophils and mononuclear cells.

*In vitro* studies have demonstrated that stimulation of lung epithelial cells with poly(I:C) elicited the secretion of multiple cytokines, chemokines, the induction of transcription factors and increased expression of TLRs [12]. In our *in vivo* model increased levels of TNF-α, IL-6, IL-8 and MCP-1 were observed in the respiratory tract, therefore a likely source of cytokines following poly(I:C) administration may be the airway epithelium. In addition, the experimental model used in this work resembles RSV infection since this respiratory virus is able to induce a profile of pro-inflammatory cytokines similar to that observed following *in vivo* poly(I:C) challenge in mice [14,16]. In fact, natural human RSV infection in children and experimental RSV inoculation in mice result in prominent local secretion of pro-inflammatory cytokines, such as TNF-α, IL-6, and CXC/CC chemokines, including IL-8, MIP-1, RANTES, and MCP-1. The coordinated actions of several of these cytokines strongly promote the recruitment and activation of neutrophils and monocytes/macrophages [27], also observed in our experimental model.

During acute viral lung infection, it is imperative that the host’s inflammatory response is tightly regulated, enabling pathogen elimination but limiting the detrimental effects of inflammation on the gas exchange. An appropriate balance of anti-inflammatory and pro-inflammatory mediators is essential for a safe and effective antiviral immune response. Thus, an excessive TNF-α/IL-8/MCP-1 response can lead to increased immunopathology, while exuberant IL-10 production can result in delayed pathogen clearance [28]. In this sense, it has been shown that TNF-α contributes to clearance of the virus during the early stages of RSV infection, which is most likely a result of the NK cell response. But continued production of TNF-α exacerbates illness and tissue injuries during the late stages of RSV infection [29]. Interestingly, recent studies demonstrate a role for IL-10 in controlling immunopathology during respiratory viral infections. Sun et al. [30] showed that IL-10 prevents immunopathology and lethal disease during acute influenza virus infection. On the other hand, IL-10 also seems to play a crucial role in controlling disease severity in RSV infection [31,32]. It was found that IL-10 deficiency during RSV challenge did not affect viral load, but led to markedly increased disease severity with enhanced weight loss, delayed recovery and a greater influx of inflammatory cells into the lung and airways and enhanced release of inflammatory mediators [33].

The preventive administration of Lr1505 reduced the production of TNF-α, IL-6, IL-8 and MCP-1 in the respiratory tract after the challenge with poly(I:C). Therefore, the reduction of these pro-inflammatory mediators could explain at least partially the reduced lung injuries in the Lr1505 treated group. Moreover, Lr1505 treatment prior to poly(I:C) challenge induced a significant increase in IL-10 in lung and serum. Consequently, IL-10 would be valuable for attenuating inflammatory damage and pathophysiological alterations in lungs challenged with the viral pathogen-associated molecular pattern poly(:IC). According to these results, Lr1505 treatment would beneficially regulate the balance between pro-inflammatory mediators and IL-10, allowing an effective inflammatory response against infection and avoiding tissue damage.

We also observed that the oral treatment with Lr1505 increased levels of IFN-γ in BAL after poly(I:C) challenge. This is in line with reports that showed improved production of IFN-γ after respiratory viral challenge in probiotic treated mice [17,21,25]. The higher levels of respiratory IFN-γ after poly(I:C) challenge in Lr1505 treated mice could be explained by the higher number of CD3^+^CD4^+^IFN-γ^+^ T cells and by an improved activation of these cells by lung DCs. In the lung, DCs are the most potent antigen presenting cells playing a central role in initiating the primary immune response. In the mouse lung, several recent studies have demonstrated the existence of two major DCs subsets identified as MHC-II^+^CD11c^+^CD11b^low^CD103^+^ (CD103^+^ DCs) and MHC-II^+^CD11c^+^CD11b^high^CD103^-^ (CD11b^high^ DCs) cells [34]. Moreover, recent studies by Furuhashi et al. [35] suggested that lung CD103^+^ DCs are more potent at eliciting Th1 and Th17 responses than CD11b^high^ DCs, whereas CD11b^high^ DCs are more efficient at evoking a Th2 response under steady state. When we analyzed lung DCs in Lr1505 treated mice after the nasal challenge with poly(I:C) we found increased levels of both CD103^+^ and CD11b^high^ DCs. Moreover, both DCs populations showed higher expression of MHC-II when compared with controls. However, IL-12 and IFN-γ were increased only in CD103^+^ DCs. Consistent our results it has been demonstrated that CD4^+^CD62L^high^DO11.10 T cells that have been primed with lung CD103^+^ DCs induced higher frequencies of CD4^+^ T cells producing IFN-γ than IL-4 [35].

An other possible source of IFN-γ in poly(I:C) challenged mice are NK cells. Similar to our work, Takeda et al., [36] showed that the oral administration of *L. plantarum* 06CC2 is able to increase IFN-γ expression in PPs and lungs. Improved respiratory IFN-γ induced by 06CC2 strain was associated with augmentation of NK cell activity and correlated with the alleviation of influenza infection in mice [36]. In addition, it was demonstrated that feeding mice with *L. pentosus* significantly enhances NK activity and that the increase in IFN-γ production by these cells did not occur through direct action of *L. pentosus* on NK cells but was dependent on IL-12 produced by intestinal CD11c^+^ DCs following the interaction between the DC and LAB [37]. Further detailed studies are required to clarify whether Lr1505 is able to increase NK cell activities and protect mice against respiratory viruses challenges.

## Conclusions

Recent evidence showed that pattern recognition receptors-mediated sensing of resident commensal microbiota in the steady state regulates the development and function of innate and adaptive immune systems in extra-intestinal sites, and prepares the host to defend against intrusion by pathogenic microorganisms [2,38]. In mice, depletion of gut microbiota by antibiotics can result in reduced surface expressions of TLR2 and TLR4 in peritoneal macrophages, and less inflammation following intraperitoneal LPS injection *in vivo*[39], indicating that intestinal microbiota can constitutively prime peritoneal macrophages in preparation for pathogen invasion. In addition, recognition of peptidoglycan from the microbiota by Nod-1 primes systemic innate immunity by enhancing the cytotoxicity of bone marrow-derived neutrophils in response to systemic infection with the bacterial pathogens, *Streptococcus pneumoniae* and *Staphylococcus aureus*[40]. Moreover, recent studies characterized the cellular and molecular mechanism by which the gut microbiota regulate respiratory tract immune defense against influenza virus infections and found that hat neomycin-sensitive bacteria in the gastrointestinal tract are required for supporting immune responses to respiratory influenza infection [41]. Collectively, these studies indicate that the gut microbiota support systemic and respiratory immunity by releasing low levels of PRR ligands in circulation.

Although our studies do not allow us to discard this mechanism for Lr1505, in the present work we propose a different mechanism influencing antiviral immune response in the respiratory tract. Our results showed that there would be a mobilization of cells from intestine and changes in cytokine profile that would be able to beneficially modulate the respiratory mucosal immunity (Figure 9). Activation of respiratory immunity by orally administered probiotics would have a complex mechanism, probably related to specific strains. Although deeper studies are needed using challenges with respiratory viruses, the results in this study suggest that Lr1505, a potent inducer of antiviral cytokines, may be useful as a therapeutic or prophylactic agent to control respiratory virus infection.

## Abbreviations

BAL, Broncho-alveolar lavage; BCA, Bicinchoninic; DCs, Dendritic cells; dsRNA, Double-stranded RNA; ELISA, Enzyme-linked immunosorbent assay; IEC, Intestinal epithelial cells; IL, Interleukin; IFN, Interferon; LAB, Lactic acid bacteria; LDH, Lactate dehydrogenase; Lr1505, *Lactobacillus rhamnosus* CRL1505; Lr1506, *Lactobacillus rhamnosus* CRL1506; NAD, Nicotinamide adenine dinucleotide; PBS, Phosphate buffer saline; RIG-I, Retinoic acid-inducible gene I; RSV, Respiratory syncytial virus; TLR3, Toll-like receptor 3; TNF, Tumour necrosis factor.

## Competing interests

The authors declare that they have no competing interests.

## Authors’ contributions

JV, EC, YT, SS and GM carried out experiments, analyzed data and performed the statistical analysis. JV, HK and SA conceived of the study, and participated in its design and coordination and helped to draft the manuscript. All authors read and approved the final manuscript.
